# Actin cytoskeleton depolymerization increases matrix metalloproteinase gene expression in breast cancer cells by promoting translocation of cysteine-rich protein 2 to the nucleus

**DOI:** 10.3389/fcell.2023.1100938

**Published:** 2023-05-15

**Authors:** Takouhie Mgrditchian, Joshua Brown-Clay, Céline Hoffmann, Tanja Müller, Liza Filali, Elena Ockfen, Xianqing Mao, Flora Moreau, Carla Pou Casellas, Tony Kaoma, Michel Mittelbronn, Clément Thomas

**Affiliations:** ^1^ Department of Cancer Research, Cytoskeleton and Cancer Progression, Luxembourg Institute of Health, Luxembourg, Luxembourg; ^2^ Department of Cancer Research, Luxembourg Centre of Neuropathology, Luxembourg Institute of Health, Luxembourg, Luxembourg; ^3^ Bioinformatics Platform, Luxembourg, Luxembourg; ^4^ Luxembourg Centre for Systems Biomedicine (LCSB), University of Luxembourg, Esch-surAlzette, Luxembourg; ^5^ Faculty of Science, Technology and Medicine (FSTM), University of Luxembourg, Esch-surAlzette, Luxembourg; ^6^ Department of Life Science and Medicine (DLSM), University of Luxembourg, Esch-surAlzette, Luxembourg; ^7^ National Center of Pathology (NCP), Laboratoire National de Santé (LNS), Dudelange, Luxembourg; ^8^ Luxembourg Center of Neuropathology (LCNP), Dudelange, Luxembourg

**Keywords:** Actin cytoskeleton, breast cancer, cysteine-rich protein 2 (CRP2), gene transcription, matrix metalloproteinases (MMPs), metastasis, serum response factor (SRF)

## Abstract

The actin cytoskeleton plays a critical role in cancer cell invasion and metastasis; however, the coordination of its multiple functions remains unclear. Actin dynamics in the cytoplasm control the formation of invadopodia, which are membrane protrusions that facilitate cancer cell invasion by focusing the secretion of extracellular matrix-degrading enzymes, including matrix metalloproteinases (MMPs). In this study, we investigated the nuclear role of cysteine-rich protein 2 (CRP2), a two LIM domain-containing F-actin-binding protein that we previously identified as a cytoskeletal component of invadopodia, in breast cancer cells. We found that F-actin depolymerization stimulates the translocation of CRP2 into the nucleus, resulting in an increase in the transcript levels of pro-invasive and pro-metastatic genes, including several members of the MMP gene family. We demonstrate that in the nucleus, CRP2 interacts with the transcription factor serum response factor (SRF), which is crucial for the expression of *MMP-9* and *MMP-13*. Our data suggest that CRP2 and SRF cooperate to modulate of MMP expression levels. Furthermore, Kaplan-Meier analysis revealed a significant association between high-level expression of SRF and shorter overall survival and distant metastasis-free survival in breast cancer patients with a high CRP2 expression profile. Our findings suggest a model in which CRP2 mediates the coordination of cytoplasmic and nuclear processes driven by actin dynamics, ultimately resulting in the induction of invasive and metastatic behavior in breast cancer cells.

## Introduction

Breast cancer is one of the most frequently diagnosed cancer types worldwide, with approximately 2.3 million new cases reported in 2020, accounting for about 11.7% of all cancer diagnoses. It is also the fifth leading cause of global cancer-related death, with approximately 685,700 deaths in 2020 (Sung et al., 2021). While localized neoplasms have a survival rate greater than 95% at 5 years, the development of invasive behavior and distant metastases drastically reduces expected survival rates to well below 30%. Current therapies primarily target tumor growth (Jin and Mu, 2015). However, breast cancer cells can disseminate later in their progression, i.e., after diagnosis (Demeulemeester et al., 2016; Yates et al., 2017), and targeting this process has a significant therapeutic potential (Leong et al., 2014).

A critical family of proteins that contribute to metastatic spread in breast cancer and other malignancies is that of the zinc-dependent endopeptidases called matrix metalloproteinases (MMPs). Originally identified as enzymes that degrade extracellular matrix (ECM) proteins, such as collagen (Gross and Lapiere, 1962), it was later found that they are also break down various other peptides, such as cytokines, cell-surface receptors, and themselves in many cases (Morrison et al., 2009; Godefroy et al., 2011; Siddhartha and Garg, 2021). Therefore, MMPs have multiple roles in cancers and participate in almost every step of the metastatic cascade, including primary tumor proliferation and survival, immune evasion, angiogenesis, basement membrane degradation, cancer cell invasion, intravasation, extravasation, and survival and growth at the distant metastatic site (Jobin et al., 2017; Winer et al., 2018).

For example, MMP-9, besides degrading collagen type IV, the primary component of the basement membrane (Kalluri, 2003), promotes tumor invasion and angiogenesis by proteolytically activating TGF-β (Yu and Stamenkovic, 2000). Although TGF-β acts as a tumor suppressor in the early stages of cancer mainly by inhibiting cell growth, it becomes a crucial tumor promoter as cancer progresses (Drabsch and ten Dijke, 2012). In addition, MMP-2, MMP-9, and MMP-14 cleave the TGF-β-binding protein 1 (LTBP-1) present in the ECM, leading to the release of ECM bound TGF-β (Dallas et al., 2002; Tatti et al., 2008), which helps establish a tumor-supportive environment. *In vivo*, mouse studies have shown that MMP-13, a “classic collagenase” almost universally upregulated across malignancies (Gobin et al., 2019), promotes mammary tumor-induced osteolysis by activating MMP-9 and increasing TGF-β signaling at the tumor-bone interface (Nannuru et al., 2010).

Various pharmacological inhibitors of MMPs have been developed, given the significance of MMPs in cancer and other diseases. However, they proved ineffective, at best, in clinical trials (Coussens et al., 2002). This was primarily due, one the hand, to the complex biology and the similarity between MMP family members (Amar and Fields, 2015), and, on the other hand, to the severe side effects of inhibitors. Significantly, some MMPs are beneficial in the context of cancer development and progression or have crucial physiological functions (Klein and Bischoff, 2011), making the use of broad-spectrum inhibitors counterproductive.

Invasive cancer cells possess specialized, actin-rich, membrane protrusions called invadopodia, which serve as a focal point for accumulating pro-invasive MMPs and other ECM-degrading enzymes (Weaver, 2006; Jacob and Prekeris, 2015; Eddy et al., 2017; Mgrditchian et al., 2021). The concentration of membrane-bound MMPs and targeted release of soluble MMPs at invadopodia result in localized and potent degradation of the ECM, which facilitates tumor cell escape from the primary tumor, and invasion into and out of the vasculature. Recently, we identified a novel and important cytoskeletal component of breast cancer cell invadopodia, namely, cysteine- and glycine-rich protein 2 (CRP2) (Hoffmann et al., 2016; Hoffmann et al., 2018). This 193-amino acid-long, two LIM domain-containing, protein directly binds to and crosslinks actin filaments, and localizes to the elongated actin core of invadopodia. Loss of CRP2 in breast cancer cells significantly decreases invadopodia formation, MMP-9 secretion, ECM degradation, and invasion *in vitro* (Hoffmann et al., 2016). Additionally, it significantly reduces metastasis in mouse breast cancer models. High levels of CRP2 is are associated with a greater risk of metastasis and reduced survival in patients (Hoffmann et al., 2016; Hoffmann et al., 2018). Interestingly, CRP2 gene expression is under the direct control of the transcription factor hypoxia-inducible factor-1 (HIF-1) (Hoffmann et al., 2018), a master driver of cancer cell adaptive responses to hypoxia (Semenza, 2016). Accordingly, CRP2 is upregulated in the hypoxic regions of breast tumors and contributes to mediating hypoxia-induced invadopodium formation in cancer cells (Hoffmann et al., 2018).

Like CRP1 and CRP3 (or muscle LIM protein), two other CRP family members, CRP2 exhibits a dual cytoplasmic and nuclear localization (Arber and Caroni, 1996; Kong et al., 1997; Chang et al., 2003). While in the cytoplasm, CRPs function as actin cytoskeleton regulatory proteins and promote the assembly of actin filament-based structures, such as stress fibers, sarcomeres, filopodia and invadopodia (Arber and Caroni, 1996; Grubinger and Gimona, 2004; Tran et al., 2005; Ma et al., 2011; Hoffmann et al., 2014; Hoffmann et al., 2016), in the nucleus, they have been proposed to act as transcriptional cofactors (Kong et al., 1997; Chang et al., 2003; Moes et al., 2013). In vascular smooth muscle cells, the cell type in which it is predominantly expressed under physiological conditions, CRP2 shuttles to shuttle to the nucleus where it physically interacts with serum response factor (SRF) and GATA transcription factors, leading to potent activation of smooth muscle gene transcription (Chang et al., 2003; Chang et al., 2007). In contrast, the presence and role of CRP2 in the nucleus of invasive breast cancer cells have not been evaluated to date.

## Results

### CRP2 shuttles to the nucleus of invasive breast cancer cells and promotes a pro-metastatic gene expression program

**FIGURE 1:**
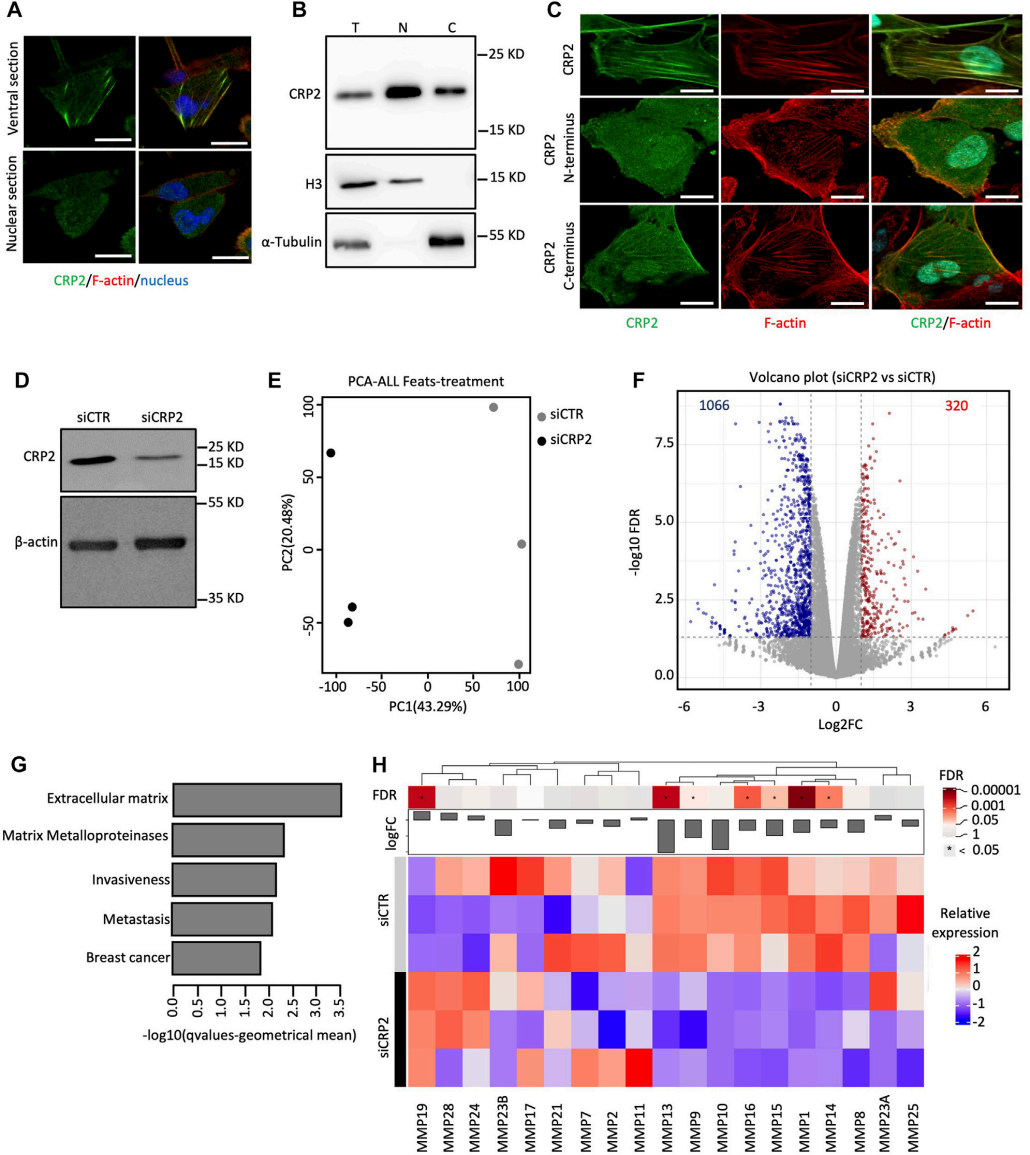
CRP2 localizes to both the cytoplasmic and the nuclear compartments and regulates gene expression in MDA-MB-231 breast cancer cells. **(A)** Subcellular distribution of endogenous CRP2 in MDA-MB-231 breast cancer cells as visualized by immunofluorescence staining and confocal microscopy. Ventral and nuclear optical sections are shown (upper and lower panels, respectively). F-actin and nucleus are labeled using fluorescent phalloidin (red) and DAPI (blue). Bars = 20 µm. **(B)** Total (T), nuclear (N) and cytoplasmic **(C)** distribution of endogenous CRP2 in MDA-MB-231 breast cancer cells as evaluated by subcellular fractionation and western blot analysis. Histone3 (H3) and *a*-Tubulin are used as nuclear and cytoplasmic markers, respectively. **(C)** Subcellular localization of CRP2-GFP, CRP2 C-terminal domain-GFP, CRP2 N-terminal domain-GFP in transfected MDA-MB-231 cells. Bars = 20 µm. **(D)** Western blot analysis showing CRP2 levels in control siRNA- and CRP2-targeting siRNA-transfected MDA-MB-231 cells subjected to RNAseq analysis. **(E)** Principal component analysis (PCA) plots for RNAseq data (three biological replicates were used for control and CRP2-depeleted cells). **(F)** Scatterplot of differentially expressed genes between control and CRP2-depeleted cells **(G)** Deregulated gene sets associated with breast cancer progression identified by gene set enrichment analysis. **(H)** K-means clustering of various matrix metalloproteases. The microscopy images presented are representative of the entire cell population. The experiments were performed in triplicate to ensure the reproducibility of the results.

We previously reported that CRP2 is upregulated in invasive breast cancer cells and is involved in the assembly and maintenance of invadopodia through its actin-bundling activity (Hoffmann et al., 2016; Hoffmann et al., 2018). In this study, we used confocal microscopy to investigate the presence of CRP2 in the nucleus of invasive breast cancer MDA-MB-231 cells. As illustrated in Supplementary Figure S1A, green fluorescence protein (GFP)-fused CRP2 was observed in the cytoplasm, primarily associated with actin-rich structures, such as actin stress fibers, and in the nucleus. The dual cytoplasmic and nuclear distribution of endogenous CRP2 was also confirmed using immunofluorescence staining (Figure 1A) and western blot analysis following subcellular fractionation (Figure 1B). Notably, the immunodetected endogenous protein exhibited a less distinct association with actin fibers compared to CRP2-GFP (Supplementary Figure S1A), a finding consistent with the previously reported difficulty in preserving the interaction between CRPs and filamentous actin when using detergents (Hoffmann et al., 2017). Additionally, we confirmed the nuclear localization of endogenous CRP2 in another triple-negative breast cancer cell line, namely, MDA-MB-468 (Supplementary Figures S1B, C), suggesting that this phenomenon is not limited to MDA-MB-231 cells.

To gain insights into how the subcellular distribution of CRP2 is regulated, we analyzed the subcellular distribution of CRP2 N- and C-terminal domains (each containing one LIM domain and representing approximately half of the full-length CRP2 protein) fused to GFP in MDA-MB-231 cells. Both constructs were observed distributing in the cytoplasmic and nuclear compartments (Figure 1C). Interestingly, in the cytoplasm, CRP2 C-terminal domain-GFP displayed a more pronounced decoration of filamentous actin than CRP2 N-terminal domain-GFP, suggesting that the F-actin binding activity of CRP2 is mainly located within its C-terminal LIM domain. Both CRP2 N- and C-terminal domains suggest that a specific LIM domain does not play a predominant role in controlling CRP2 nuclear translocation. The relatively small size of the CRP2 constructs (<40 kD) may allow them to passively diffuse across the nuclear pores. The nuclear translocation dynamics of the N-terminal and C-terminal domains were evaluated using fluorescence recovery after photobleaching (FRAP). After photobleaching the nucleus, CRP2 N-terminal domain-GFP fluorescence recovered rapidly with kinetics almost identical to those obtained for GFP alone (Supplementary Figures S2A, B). In contrast, CRP2 C-terminal domain-GFP fluorescence recovered at a significantly lower rate, similar to full-length CRP2-GFP. This suggests that the CRP2 C-terminal LIM domain reduces CRP2 nuclear translocation by mediating CRP2 interaction with filamentous actin in the cytoplasm (see further below). Although CRP2 lacks a nuclear export signal motif (as evaluated by prediction tools), leptomycin B treatment consistently resulted in slight increased amounts of nuclear CRP2 (Supplementary Figure S2C), suggesting that CRP2 nuclear export may be regulated by a nuclear partner interacting with exportin 1/CMR1.

As CRP2 was previously reported to function as a transcriptional cofactor in smooth muscle cells (Chang et al., 2003), we investigated whether CRP2 regulates gene expression in breast cancer cells. We used RNA sequencing (RNA-seq) to identify changes in gene expression caused by transient knockdown of CRP2 in MDA-MB-231 cells (Figure 1D). Principal component analysis and correlation analysis revealed a distinct transcriptional profile in CRP2-depleted cells compared to control cells (Figure 1E). The analysis of differentially expressed genes showed that targeting CRP2 led to a significant downregulation of 1,066 genes and an upregulation of 320 genes with an FDR of <0.05 and FC > 2 (Figure 1F). Gene set enrichment analysis identified deregulated gene sets associated with the ECM, MMPs, invasiveness, metastasis, and breast cancer (Figure 1G and Supplementary Figure S3; Supplementary RNAseq data set). These results are highly consistent with the previously reported role of CRP2 in breast cancer progression (Hoffmann et al., 2016; Hoffmann et al., 2018). The most upregulated pathways included RNA processing, DNA processing and cell cycle (Supplementary Figure S3). In this study, our focus was on MMPs, which, as mentioned in the introduction, play a critical role in ECM degradation and invasion. Notably, depletion of CRP2 resulted in altered expression of seven *MMPs*, with six of them being downregulated (Figure 1H).

**FIGURE 2:**
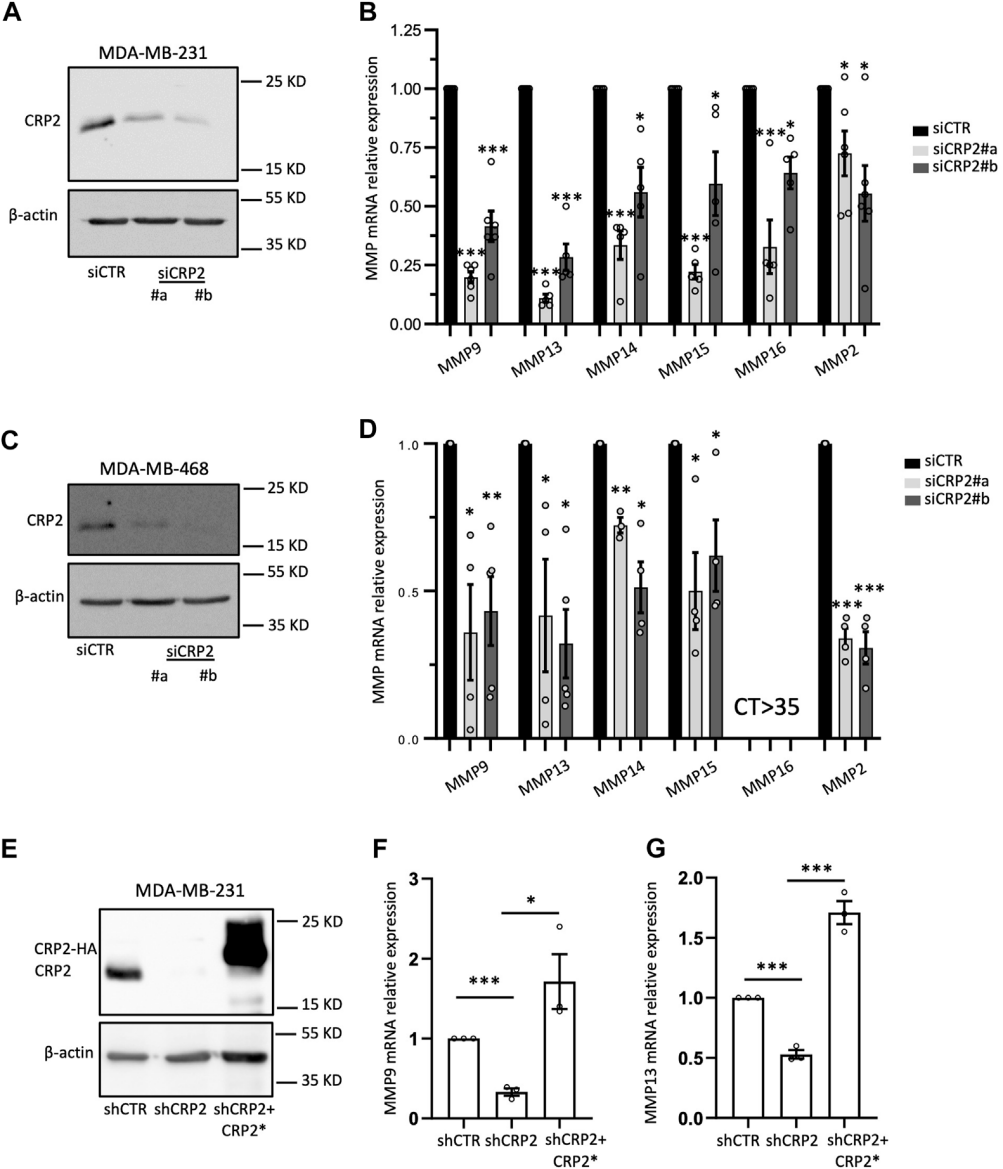
CRP2 upregulates MMP transcript levels in invasive breast cancer cell lines. **(A–D)** MDA-MB-231 and MDA-MB-468 cells were transfected with non-targeting control siRNAs or two different CRP2-targeting siRNAs (siCRP2#a and #b). The blots on the left show CRP2 protein levels (western blot) in MDA-MB-231 **(A)** and MDA-MB-468 cells **(C)** 68 h after transfection. The charts on the right show the transcript level for several MMP genes in MDA-MB-231 **(B)** and MDA-MB-468 cells **(D)** as evaluated by real-time RT-qPCR. **(E)** Western blot showing CRP2 protein levels in MDA-MB-231 cell-derived cell lines stably expressing a control non-targeting shRNA (shCTR) and CRP2-targeting shRNA (shCRP2), and in a “rescue” cell line (shCRP2+CRP2*) overexpressing a HA-fused, shRNA-resistant CRP2, variant (CRP2*). **(F,G)** MMP-9 **(F)** and MMP-13 **(G)** transcript levels in the shCTR, shCRP2 and shCRP2+CRP2* MDA-MB-321 cell lines as evaluated by real-time RT-qPCR. Results are expressed as the average ±SEM of at least three independent experiments (open circles); * denotes a *p*-value less than 0.05, ** a *p*-value less than 0.01, *** a *p*-value less than 0.001 (two-tailed Student’s t test).

To expand on our RNA-seq analysis and confirm the role of CRP2 in regulating pro-invasive MMP expression in breast cancer cells, we knocked down CRP2 in MDA-MB-231 cells using two different siRNAs (Figure 2A) and assessed the expression of several *MMPs* associated with breast cancer progression, namely, *MMP-2*, *MMP-9*, *MMP-13*, *MMP-14*, *MMP-15* and *MMP-16*, using real-time quantitative reverse transcription PCR (RT-qPCR). As illustrated in Figure 2B, the mRNA levels of all six MMPs were significantly decreased in CRP2-depleted cells as compared to control cells. We also extended our investigations to another aggressive breast cancer cell line, MDA-MB-468. Once again, the knockdown of CRP2 significantly reduced the mRNA expression levels of all the MMPs analyzed, except *MMP-16*, whose basal expression level in MDA-MB-468 cells was not significant (CT > 35) (Figures 2C, D). Together, our findings suggest that, in addition to its cytoplasmic function in regulating actin cytoskeleton organization and dynamics at invadopodia, CRP2 induces a pro-invasion gene expression program and upregulates the expression of *MMPs* in breast cancer cells.

### Actin dynamics modulate CRP2 nuclear abundance and *MMP-9* and *MMP-13* transcript levels

In the subsequent sections discussing the molecular mechanism underlying CRP2-driven *MMP* expression, we focused on two *MMPs* that were significantly downregulated following CRP2 knockdown in both MDA-MB-231 and MDA-MB-468 cells, namely, *MMP-9* and *MMP-13* (Figures 2A–D).

**FIGURE 3:**
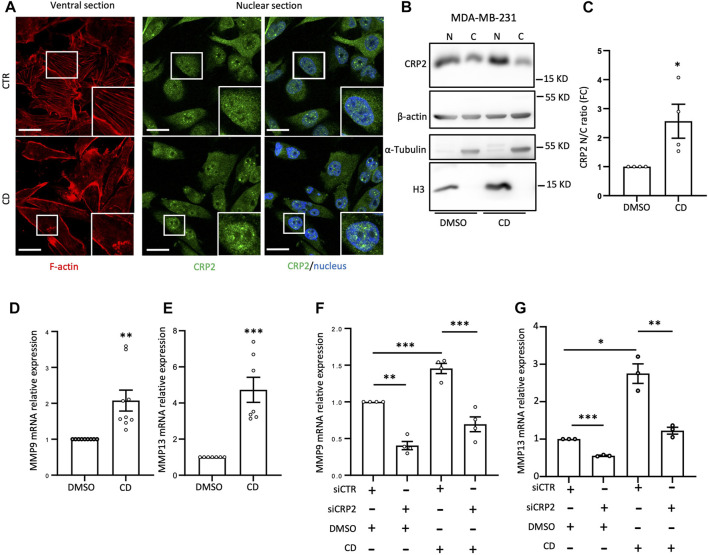
Actin depolymerization increases MMP-9 and MMP-13 transcript levels by promoting CRP2 nuclear translocation. **(A)** Effects of control (DMSO) and cytochalasin-D (CD) treatment on F-actin-based structures and nuclear abundance of endogenous CRP2 in MDA-MB-231 cells as visualized by phalloidin staining (red) and immunofluorescence staining (green), respectively. Nuclei are labeled with DAPI (blue). The microscopy images presented are representative of the entire cell population. **(B)** Effects of control (DMSO) and cytochalasin-D (CD) treatment on endogenous CRP2 subcellular distribution in MDA-MB-231 cells as evaluated by subcellular fractionation and western blot analysis. Histone3 (H3) and *a*-Tubulin are used as nuclear and cytoplasmic markers, respectively. **(C)** CRP2 nuclear/cytoplasmic ratio quantified from four independent western blot analyses, similar to the one shown in **(B)**. **(D,E)** MMP-9 **(D)** and MMP-13 **(E)** transcript levels in control and CD-treated cells. **(F,G)** MMP-9 **(F)** and MMP-13 **(G)** transcript levels in control and CD-treated cells combined with non-targeting control siRNA or CRP2-targeting siRNA treatment. Results are expressed as the average ±SEM of at least three independent experiments (open circles); * denotes a *p*-value less than 0.05, ** a *p*-value less than 0.01, *** a *p*-value less than 0.001 (two-tailed Student’s t test).

To further investigate the causal relationship between CRP2 and MMP gene expression, we utilized a modified MDA-MB-231 cell line stably expressing CRP2-targeting shRNAs with low residual CRP2 protein levels, and a derived “rescue” cell line overexpressing a shRNA-resistant CRP2 coding sequence (Hoffmann et al., 2016) (Figure 2E). Consistent with our previous findings, *MMP-9* and *MMP-13* mRNA levels were significantly decreased in shCRP2 MDA-MB-231 cells compared to the control cells expressing a non-targeting shRNA (Figures 2F, G). The expression of shRNA-resistant CRP2 in the rescue cell line was sufficient to restore a higher expression of both *MMP* transcripts, supporting that the effects of CRP2-targeting siRNAs and shRNA on *MMP* gene expression are specifically due to CRP2 depletion. Interestingly, the high amount of recombinant CRP2 protein expressed by the rescue cell line (compared to the lower amount of endogenous CRP2 protein in the parental cell line; Figure 2F) did not fully translate into a proportional increase in MMP mRNA levels (Figures 2F, G), suggesting an additional layer of regulation. Based on the above-described FRAP data, we hypothesized that the nuclear abundance and associated gene-regulatory activity of CRP2 in breast cancer cells are modulated by the actin polymerization state, i.e., the relative amounts of G- and F-actin. To test this hypothesis, MDA-MB-231 and MDA-MB-468 cells were treated with the actin-depolymerizing drug cytochalasin D (CD) and evaluated for the subcellular distribution of endogenous CRP2. Depolymerization of actin filaments in CD-treated cells was evidenced by decreased actin stress fibers as vizualized using fluorescent phalloidin staining (Figure 3A; Supplementary Figure S5A). This effect was associated with a noticeable increase in CRP2 nuclear amounts in immunofluorescence assays. The translocation of CRP2 upon CD treatment was confirmed using subcellular fractionation and western blot analysis (Figure 3B; Supplementary Figure S5B). Quantitative analysis revealed that actin cytoskeleton depolymerization resulted in an approximately 2.5-fold increase in the nuclear/cytoplasmic CRP2 ratio in MDA-MB-231 and MDA-MB-468 cells (Figure 3C; Supplementary Figure S5C). To assess whether the actin cytoskeleton depolymerization-dependent increase of the CRP2 nuclear fraction translated into an upregulation of *MMP-9* and *MMP-13* mRNA levels, we used real-time RT-qPCR. Remarkably, CD treatment increased *MMP-9* and *MMP-13* mRNA levels by several folds in both cell lines (up to 10-fold in MDA-MB-468) compared to the respective mock-treated controls (Figures 3D, E; Supplementary Figures S5D, E). To ascertain that the effects of CD treatment on *MMP-9* and *MMP-13* transcript levels were mediated by CRP2, the above analysis was repeated using CRP2-depleted MDA-MB-231 cells. As shown in Figures 3F, G, CRP2 knockdown abolished the actin cytoskeleton depolymerization-mediated upregulation of both *MMPs*. Altogether our data suggest that actin dynamics regulate CRP2 nuclear amounts, thereby controlling *MMP-9* and *MMP-13* expression.

### Serum response factor interacts with CRP2 and is required for *MMP-9* and *MMP-13* expression in breast cancer cells

**FIGURE 4:**
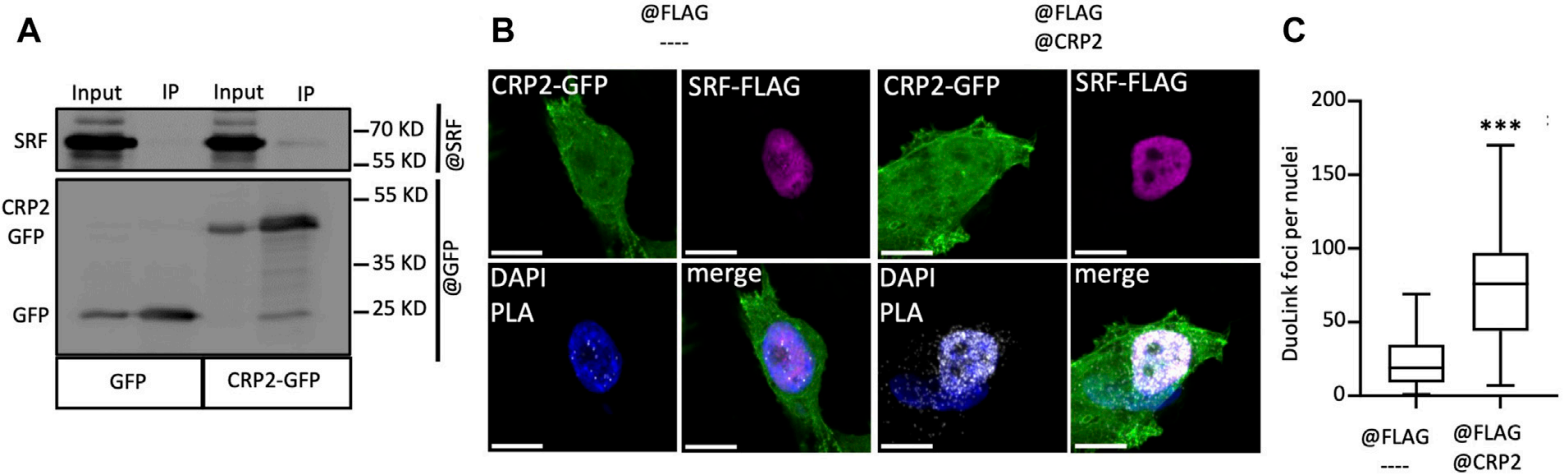
CRP2 interacts with the transcription factor SRF in breast cancer cells. **(A)** Protein extracts of GFP and CRP2-GFP expressing MDA-MB-231 cells were subjected to immunoprecipitation using anti-GFP-nanobodies covalently bound to magnetic agarose beads (GFP-Trap^®^). Input and bound fractions (IP) were probed for SRF and GFP. **(B)** PLA images where the PLA signal (white foci) indicates close proximity between CRP2-GFP (green) and SRF-FLAG (purple) in MDA-MB-231 cells (right panels). Unspecific PLA signal was evaluated by omitting anti-CRP2 antibodies (left panels). Nuclei were counterstained with DAPI (blue). Bars = 10 µm. **(C)** Quantitative analysis of Duolink^®^ PLA foci in the nuclei of MDA-MB-231 cells. The center lines of the box-and-whisker diagram denotes the median value (50th percentile), while the upper and lower boxes represent the first and third quartiles, respectively. The whiskers extend to the minimum and maximum values of the data set. Data originate from 4 independent experiments, including at least 77 randomly selected cells. *** a *p*-value less than 0.001 (two-tailed Student’s t test). The microscopy images presented are representative of the entire cell population.

**FIGURE 5:**
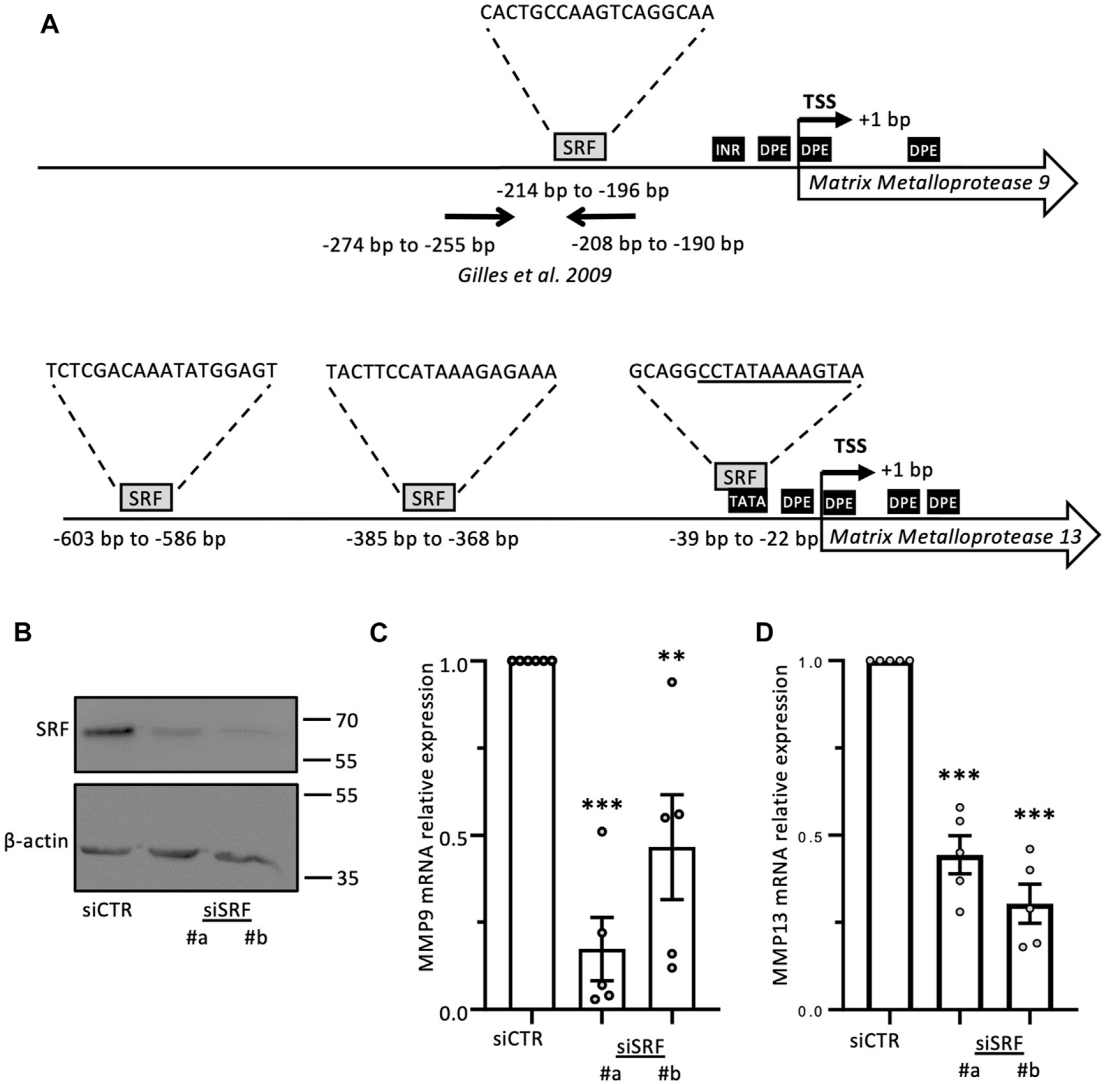
MMP-9 and MMP-13 transcript levels are regulated by SRF. **(A)** Schematic representation of *MMP-9* and *MMP-13* proximal promoter regions. Regulatory promoter elements including TATA-box, INR and DPE, as well as position (relative to the transcription start site, TSS) and sequence of predicted binding sites for SRF are indicated. Further information is provided in Supplementary Table S3. **(B–D)** Effects of SRF knockdown, as verified by western blot analysis **(B)**, on MMP-9 and MMP-13 transcript levels in MDA-MB-231 cells (C and D, respectively). Results are expressed as the average ±SEM of at least three independent experiments (open circles); ** a *p*-value less than 0.01, *** a *p*-value less than 0.001 (two-tailed Student’s t test).

**FIGURE 6:**
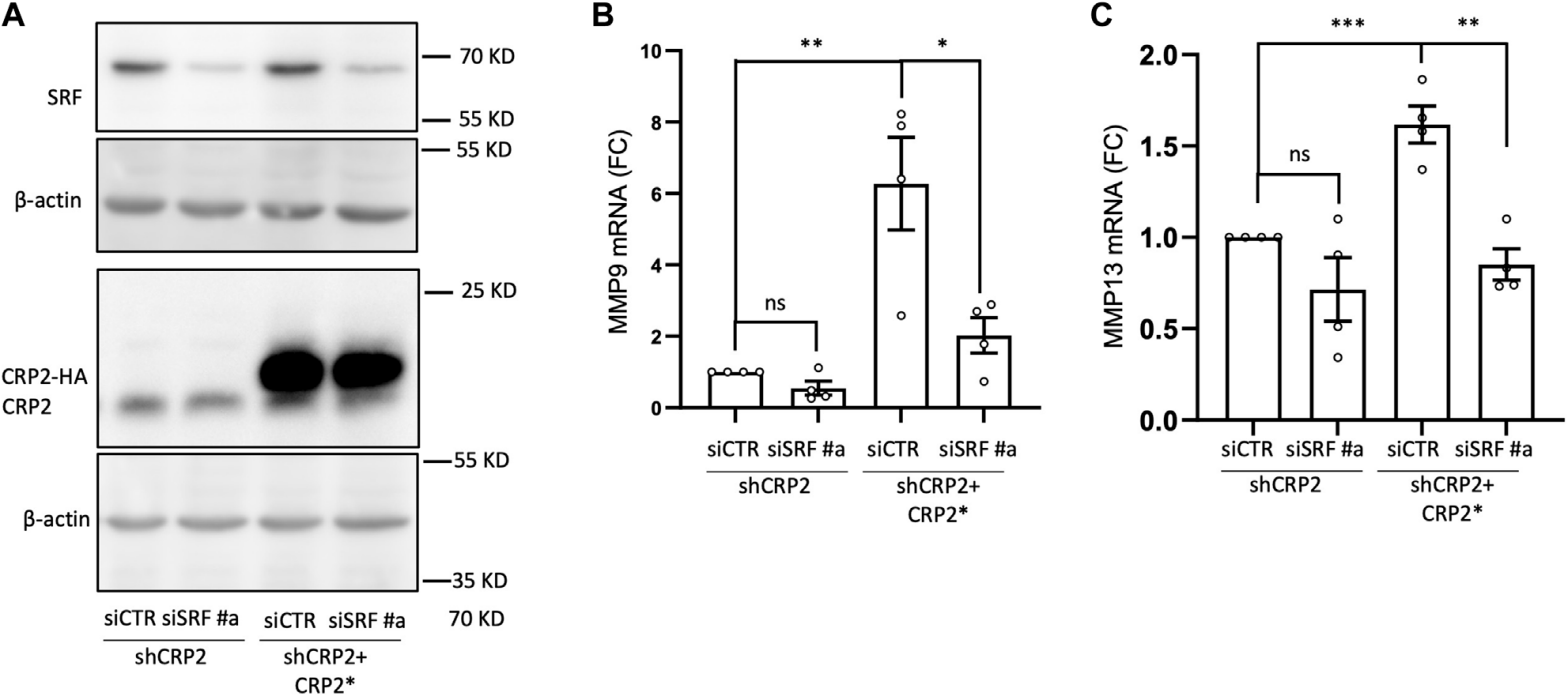
Interdepence of SRF and CRP2 in regualting MMP-9 and MMP-13 transcript levels. **(A)** Western blot analysis of SRF and CRP2 (or HA-CRP2) protein levels in MDA-MB-231 cell-derived shCRP2 and shCRP2+CRP2* cell lines, 48 h following their transfection with non-targeting or SRF-targeting siRNAs (siCTR and siSRF, respectively). **(B,C)** MMP-9 **(B)** and MMP-13 **(C)** transcript levels in cells described in **(A)** as evaluated by real-time RT-qPCR. Results are expressed as the average ±SEM of at least three independent experiments (open circles); ^ns^ non significant, * denotes a *p*-value less than 0.05, ** a *p*-value less than 0.01, *** a *p*-value less than 0.001 (two-tailed Student’s t test).

The CRP family members mostly consist of two LIM domains, which mediate protein-protein interactions (Schmeichel and Beckerle, 1994), and have not been reported to function as transcription factors. Yet, CRP2 has been shown to function as a transcription cofactor that associates with transcription factors, particularly SRF, to promote the upregulation of smooth muscle genes during early differentiation of smooth muscle cells (Chang et al., 2003). Interestingly, a role for SRF in controlling MMP gene expression has previously been proposed in the context of megakaryocyte migration and tissue damage in pulmonary lymphangioleiomyomatosis (Zhe et al., 2003; Gilles et al., 2009). To determine if CRP2 interacts with SRF in invasive breast cancer cells, CRP2-GFP was expressed in MDA-MB-231 cells and immunoprecipitated using GFP-Trap, and the pull-down was probed with an anti-SRF antibody. As shown in Figure 4A, SRF was detected in the bound fraction but not when the cell lysate was prepared from control, GFP-expressing, MDA-MB-231 cells. To validate CRP2-SRF interaction with an alternate approach and determine in which subcellular compartment(s) this interaction occurs, a proximity ligation assay (PLA) was employed. CRP2-GFP-expressing MDA-MB-231 cells were transfected with an SRF-FLAG expressing construct, and PLA was performed using anti-CRP2 and anti-FLAG monoclonal antibodies. Many strong fluorescent signals corresponding to PLA foci were produced in the nucleus of cells, while no or only a few fluorescent signals were present in the cytoplasm (Figures 4B, C). In contrast, an anti-FLAG antibody alone detected a much lower number of PLA foci in a negative control. Together, the above results support that CRP2 and SRF interact in the nucleus of breast cancer cells and suggest that SRF is a transcription factor critical to *MMP* expression. Supporting this hypothesis, SRF binding sites are located in the proximal promoter region of both *MMP-9* and *MMP-13* (Figure 5A; Supplemetary Table S3). To confirm the regulatory role of SRF in MMP gene expression in breast cancer cells, we used two different SRF-targeting siRNAs to knock down SRF and quantified the levels of *MMP-9* and *MMP-13* mRNAs using real-time RT-qPCR. As shown in Figures 5B–D, the depletion of SRF led to a significant decrease in both *MMP* transcript levels. To further assess the interdependence between SRF and CRP2 in the regulation of *MMP* expression, we repeated the analysis in the shCRP2 MDA-MB-231 cell line, characterize by minimal residual CRP2 expression (Figures 2E–G). Interestingly, SRF knockdown failed to induce a statistically significant decrease in *MMP-9* and *MMP-13* mRNA levels in shCRP2 MDA-MB-231 cells (Figures 6A–C). In contrast, the effect of the depletion of SRF on the expression levels of both *MMP*s was restored in the rescue MDA-MB-231 cell line expressing high amounts of CRP2. This suggests that SRF and CRP2 function interdependently (see further below).

### High *SRF* expression correlates with shorter overall and distant metastasis-free survival in patients with high *CRP2* expression

**FIGURE 7:**
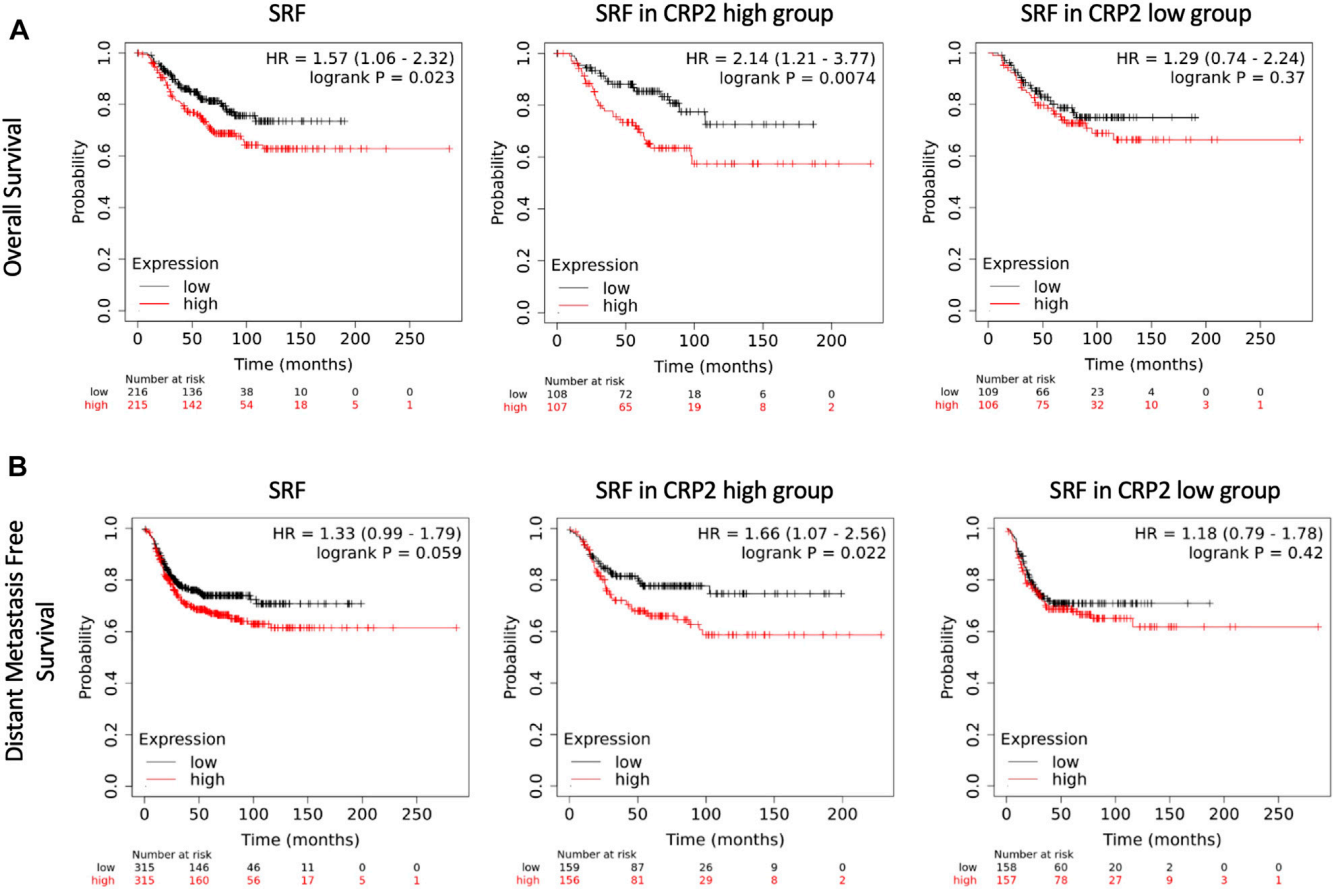
Kaplan-Meier survival analyses in relation to SRF expression in basal-like breast carcinoma. The upper **(A)** and lower **(B)** panels show overall and distant metastasis free survival, respectively. Left panels denote survival in the total population of basal-like breast cancer patients (PAM50), while middle and right panels denote survival in basal-like breast cancer patients with high and low CRP2 expression profile, i.e., above and below median, respectively. The patient samples, hazard ratio with 95% confidence interval, and *p*-value (Logrank test) are displayed on each chart.

We previously reported that higher *CRP2* expression is correlated with significantly shorter overall and distant metastasis-free survival (OS and DMFS, respectively) in basal-like breast cancer (Hoffmann et al., 2016; Hoffmann et al., 2018), a molecular subtype of breast cancer with aggressive behavior and in which CRP2 is upregulated (Hu et al., 2006; Hoffmann et al., 2016). The clinical significance of *SRF* expression in basal breast cancer was evaluated by Kaplan-Meier analysis using publicly available transcriptomics data sets (Gyorffy et al., 2010). When all the patients from the basal-like subtype (PAM50) (Parker et al., 2009) were included (*n* = 431), Kaplan-Meier analysis revealed a significant correlation between high *SRF* expression (above median) and shorter OS (HR = 1.57, log-rank *p* = 0.023), while *SRF* expression and DMFS did not correlate in a statistically significant manner (HR = 1.33, log-rank *p* = 0.059) (Figures 7A, B; left panels). Remarkably, when the analysis was restricted to patients with a high *CRP2* expression profile (i.e., above median), the correlation between high *SRF* expression (above median) and shorter OS increased significantly with a hazard ratio = 2.14 (log-rank *p* = 0.0074) and a significant correlation between high *SRF* expression (above median) and DMFS was established (HR = 1.66, log-rank *p* = 0.022) (Figures 7A, B; middle panels). Upper quartile survival for patients with low and high SRF expression was 108 and 44.4 months, respectively, for OS, and 102.6 and 29.2 months, respectively, for DMFS. A significant correlation between *SRF* expression and OS or DMFS was lost when the analysis was restricted to patients with a low *CRP2* expression profile (i.e., below median) (Figures 7A, B; right panels). These findings further support that CRP2 and SRF function interdependently and cooperate in driving the expression of pro-invasive MMPs in breast cancer cells, exacerbating the clinical outcome of patients by enhancing metastasis.

## Discussion

**FIGURE 8:**
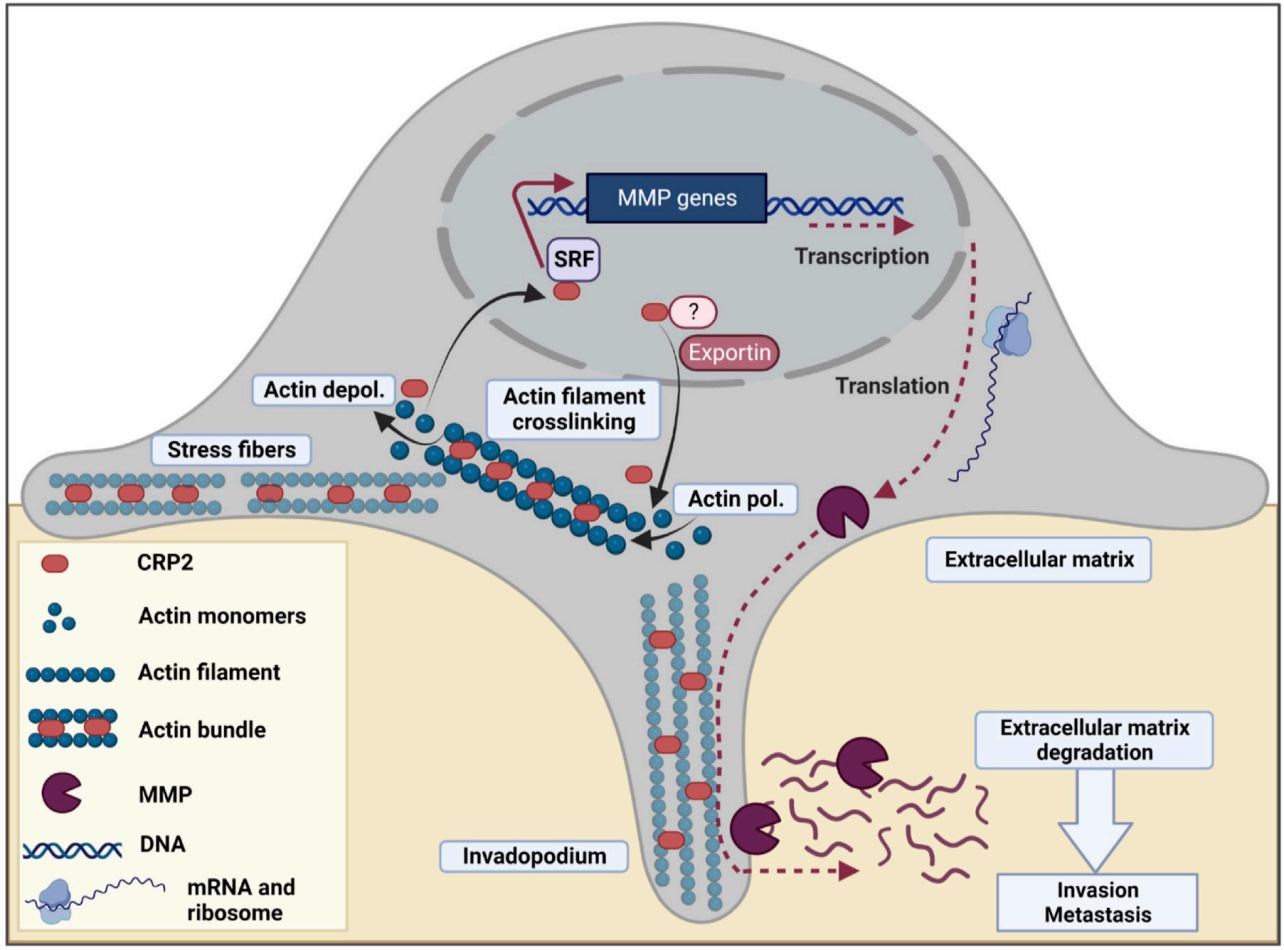
Model for the dual cytoplasmic and nuclear roles of CRP2 in breast cancer invasion and metastasis. In breast cancer cells, CRP2 exhibits dual cytoplasmic and nuclear localization. In the cytoplasm, CRP2 binds to and crosslinks actin filaments, and contributes to forming and maintaining pro-invasive actin-rich structures, such as invadopodia (Hoffmann et al., 2016; Hoffmann et al., 2018). In the nucleus, CRP2 associates with SRF and upregulates the expression of pro-invasive genes, including several MMPs known for their ability to degrade the extracellular matrix and facilitate the early steps of invadopodia formation, such as MMP-14/MT1-MMP (Ferrari et al., 2019). The relative distribution of CRP2 in the cytoplasmic and nuclear compartments is modulated by actin dynamics. Upon actin filament depolymerization, e.g., during disassembly of aging invadopodia, free CRP2 is released and shuttles to the nucleus. The presence of a nuclear localization signal (NLS) in the N-terminal region of CRP2 may facilitate the trafficking of CRP2 to the nucleus. Although CRP2 also lacks a nuclear export signal (NES), the nuclear amount of CRP2 increases upon leptomycin B treatment, suggesting that CRP2 interacts with a partner that is regulated by exportin 1/CMR1.

We previously reported that CRP2 acts as an F-actin-binding and -bundling protein in the cytoplasm and localizes to stress fibers and invadopodia (Hoffmann et al., 2016). Functional analysis revealed that CRP2 promotes invadopodium formation and maturation and is critical for polarizing the secretion of pro-invasive MMPs, such as MMP-9, as well as 3D matrix degradation and invasion (Hoffmann et al., 2016; Hoffmann et al., 2018). The present study establishes that CRP2 is also present in the nucleus of breast cancer cells and drives a pro-invasive and -metastatic gene expression program. This suggests an attractive model in which CRP2 potentiates breast cancer cell invasive behavior through complementary and interdependent cytoplasmic and nuclear mechanisms (Figure 8).

Our findings suggest that an increase in the G-actin/F-actin ratio triggers the translocation of CRP2 to the nucleus in breast cancer cells. This observation is in line with previous studies demonstrating that the distribution of CRP2 in smooth muscle cells is regulated by the actin polymerization state, which is largely influenced by the abundance of actin stress fibers (Chang et al., 2003; Kihara et al., 2011). Specifically, the levels of nuclear CRP2 tend to rise when the actin cytoskeleton is depolymerized. Importantly, we also observed that the accumulation of CRP2 in the nucleus of breast cancer cells is associated with an increase in the expression of *MMP-9* and *MMP-13* transcripts. Moreover, our results indicate that the upregulation of *MMP-9* and *MMP-13* is directly mediated by nuclear CRP2, as knocking down CRP2 abolishes the increase in transcript levels induced by actin cytoskeleton depolymerization.

Other actin-binding proteins have been previously characterized as regulators of gene transcription mediated by actin dynamics, with myocardin-related transcription factor-A (MRTF-A), also known as Megakarocytic Acute Leukemia (MAL), being the most extensively studied (Miralles et al., 2003; Olson and Nordheim, 2010). Unlike CRP2, which is an F-actin-binding protein, MAL is a G-actin-binding protein released from actin monomers and accumulates into the nucleus upon stimulation of actin polymerization when G-actin is recruited into actin filaments. Conversely, we found that actin depolymerization increases the nuclear abundance of CRP2.

Our data suggest that the dynamics of CRP2 in breast cancer are regulated by a complex regulatory mechanism that involves at least three components. First, the C-terminal domain of CRP2, which primarily consists of the second LIM domain, promotes the interaction of CRP2 with F-actin-based structures, such as stress fibers (Figure 1C) and invadopodia (Hoffmann et al., 2016; Hoffmann et al., 2018), while reducing the nuclear amount of CRP2. This observation is consistent with previous findings showing that the C-terminal LIM domain of CRP3, a close relative of CRP2, is responsible for F-actin-binding activity, while the N-terminal LIM domain drives protein dimerization and is required for actin-bundling activity (Hoffmann et al., 2014). Second, the N-terminal moiety of CRP2 contains a nuclear localization signal (NLS) (Weiskirchen et al., 1995; Wang et al., 2003), which may facilitate the translocation of free CRP2 to the nucleus. Third, the export of CRP2 from the nucleus seems to be facilitated, at least in part, by exportin 1/CMR1. Notably, CRP2 lacks a NES motif, suggesting that its translocation to the cytoplasmic may involve a nuclear partner that has yet to be identified.

Both CRP2 and SRF were previously reported to promote MDA-MB-231 cell invasion *in vitro* and metastasis in experimental metastasis assays (Medjkane et al., 2009; Hoffmann et al., 2016; Hoffmann et al., 2018). Our immunoprecipitation and PLA data support the interaction between CRP2 and SRF in breast cancer cells. Similar to CRP2 depletion, the depletion of SRF significantly reduced *MMP-9* and *MMP-13* transcript levels. However, it has no significant effect on the residual expression of *MMP-9* and *MMP-13* in CRP2-depleted cells, further suggesting that SRF and CRP2 functionally interact and cooperate to upregulate *MMP* expression. Consistent with these findings, a strong correlation between SRF expression and overall survival or distant metastasis-free survival is only observed in patients with higher CRP2 expression levels. Previously, CRP2 was characterized as a transcription cofactor that interacts with SRF and enhances SRF-dependent activation of smooth muscle genes during muscle cell differentiation (Chang et al., 2003; Chang et al., 2007). Our data support the existence of a CRP2-SRF in breast cancer.

In a previous study, we established that the hypoxia-inducible factor-1 (HIF-1) pathway, a key mediator of the response to hypoxia, directly controls CRP2 transcriptional activation, and CRP2 expression is accordingly upregulated in hypoxic areas of breast tumors (Hoffmann et al., 2018). Hypoxia is a well-recognized driver of tumor aggressiveness and metastasis, and is associated with poor clinical outcomes in various malignancies, including breast cancer (Gilkes et al., 2014; Walsh et al., 2014; Rankin and Giaccia, 2016; Semenza, 2016). Cellular responses to hypoxia mainly result from significant transcriptional changes initiated by master transcription factors, such as HIF-1. Our data suggest that CRP2 could mediate the hypoxia-induced transactivation of pro-invasion and -metastasis genes, such as MMP genes. Hypoxia and the HIF-1 pathway have been shown to upregulate several members of the *MMP* family in breast cancer, including *MMP-2* and *MMP-9*, (Krishnamachary et al., 2006; Munoz-Najar et al., 2006; Choi et al., 2011). TGF-β, another important feature of the tumor microenvironment and late-stage enhancer of cancer progression (Drabsch and ten Dijke, 2011), also upregulates *CRP2* expression (Herrmann et al., 2006). Therefore, we propose that CRP2 is an essential component of the aggressive tumor phenotype induced by either of these features of the tumor microenvironment.

The findings reported here are particularly compelling, the failures of MMP inhibitors in the clinic (Coussens et al., 2002; Amar and Fields, 2015). Targeting the metastatic machinery of the cancer cell has shown pre-clinical promise (Leong et al., 2014; Stoletov and Lewis, 2015; Gandalovicova et al., 2017), particularly in the pre-to peri-surgical resection window. However, conventional neo-adjuvant therapy can increase cancer cell invasion, intravasation and metastatic dissemination (Karagiannis et al., 2017). Additionally, the surgical procedure can enhance breast cancer cells’ metastatic seeding (Katharina, 2011; Tohme et al., 2017). Therefore, there is a strong rationale for targeting cancer cells’ invasive or metastatic ability. CRP2 emerges as a potential therapeutic target for breast cancer with a poor prognosis due to its dual role in promoting invadopodia formation in the cytoplasm and upregulating pro-invasive genes, including *MMPs*, in the nucleus. Moreover, the importance of SRF in promoting cancer-associated processes, such as cell proliferation, invasion, and metastasis, is increasingly recognized (Azam et al., 2022). However, targeting SRF activity selectively in diseased cells remains a significant challenge. Targeting CRP2 could offer an opportunity to selectively target SRF in breast cancer cells.

## Material and methods

### Cell culture

MDA-MB-231 cell line was purchased from ATCC. MDA-MB-468 cell line was authenticated and checked for cross-contamination through STR profiling analysis (Eurofins). Both cell lines were regularly tested for *mycoplasma* contamination and cultured in complete DMEM medium in a standard tissue culture incubator (21% O_2_, 5% CO_2_, 37°C), following ATCC recommendations. MDA-MB-231 wild type cells were used to generate the control (shCTR), CRP2 knockdown (shCRP2) and CRP2 rescue (shCRP2+CRP2*) cell lines, as previously described (Hoffmann et al., 2016). Cells were cultured in DMEM supplemented with 10% (v/v) fetal bovine serum (FBS, Gibco), 100 U/mL penicillin and 0.1 mg/mL streptomycin (Sigma-Aldrich). For Cytochalasin D (CD) treatment, cells were treated for 16 h with 0.5 µM of CD (Enzo; BML-T109-0001). For leptomycin B treatment, cells were treated for 16 h with 20 nM of leptomycin B (SIGMA; L2913-.5UG). CMR1/Exportin 1 was used as a conrol for letpomycin treatment (Rahmani and Dean, 2017).

### RNA sequencing

For RNA sequencing, total RNA was extracted from siCTR and siCRP2#a transfected MDA-MB-231 cells using RNeasy Mini kit (Qiagen) according to manufacturer’s instructions. RNA purity was assessed using the Fragment Analyzer Systems, 1 µg of RNA with an RNA quality number of 10 was used for RNA sequencing. TruSeq stranded mRNA library preparation on 6 samples (3 replicates per group) was performed according to Illumina standard protocol. The 6 libraries prepared were quantified with Qubit dsDNA kit HS and the size for each library was estimated using the HS-NSG Fragment Analyzer kit. The 6 libraries were pooled at 10 nM and paired-end of 75 bp reads was performed using mid output flow cell configuration. RNA sequencing read quality was assessed with FastQC using the online available tool, http://www.bioinformatics.babraham.ac.uk/projects/fastqc/. Next, reads were aligned to the Human reference genome (GRCh38 assembly) using STAR (Dobin et al., 2013) using the -*-quantMode GeneCounts* parameter to obtain an integrative matrix of raw counts for each gene. These initial data processing steps were handled by the Snakemake workflow management system (Molder et al., 2021). Raw counts of 58,396 genes from the 6 samples was imported into R environment (4.2.1) for gene expression analysis. First, genes not expressed in more than 50% of samples in one group were discarded. Next, counts were converted to counts per millions (CPM) (edgeR version 3.32.1) enabling comparison of samples with different library sizes. After log2 transformation of CPM, principal component analysis (PCA) was performed for dimensionality reduction and quality assessment. Differentially expression analysis of siCTR and siCRP2 cells was made through exactTest function of edgeR package. Genes with FDR <5% and absolute log_2_FC ≥ log_2_ (1.5) were considered as differentially expressed. RNA-Seq data is accessible under GEO accession number GSE199822.

### RNA extraction and real-time quantitative RT-PCR

Total RNA was extracted from cells using RNeasy Mini kit (Qiagen) according to manufacturer’s instructions. Total RNA (2.4 μg) was converted to cDNA using iScript™ Advanced cDNA synthesis Kit (Bio-Rad). Real-time quantitative RT-PCR was performed using the 2X Takyon for SYBR assay-Low ROX (Eurogentec) in an Applied Biosystems Quant Studio 3 Real-Time PCR System (Thermo Fisher Scientific). Relative gene expression values were calculated using the ΔΔCt method and normalized to the ribosomal protein S18 (RPS18). The sequence of primers is given in Supplementary Table S1.

### Immunofluorescence staining and confocal microscopy

Cells seeded in ibidi µ-slide chambers were fixed with 4% paraformaldehyde, permeabilized with 0.1% Triton X-100 and blocked with 2% bovine serum albumin (BSA) and 2% fetal bovine serum (FBS) in PBS. Cells were incubated with primary antibody in 2% BSA overnight at 4°C, washed 3 times in PBS and labeled with Acti-stain™ 555 or 670 phalloidin (100 nM, Cytoskeleton) in 2% BSA for 30 min at room temperature. Cells were washed 3 times in PBS, stained with DAPI (100 ng/mL, SIGMA) in PBS for 5 min and, washed in PBS and mounted in ibidi Mounting Medium. Imaging was performed with a laser scanning confocal microscope (LSM880 FastAiry, Carl Zeiss) equipped with a ×63/1.4 numerical aperture (NA) oil immersion Plan-Apochromat objective. All pictures were acquired with multitrack configuration with a confocal optical section set at 1 μm thickness. The nuclear/cytoplasmic ratio were calculated from z-stack images encompassing the entire cell volume using ImageJ. Three independent experiments were performed for MDA-MB-231 and MDA-MB-468 cells. A total of 72 and 86 MDA-MB-231 cells were analyzed for control (CTR) and CD conditions, respectively. A total of 69 and 65 MDA-MB-468 cells were analyzed for control (CTR) and CD conditions, respectively. For FRAP analysis, cells were transfected with the indicated constructs and plated in ibidi µ-slide chambers, the time-lapse was set to acquire one image every 30 s for a period of 10 min. The 488 nm laser was applied on an ellipsoid region of interest (ROI) covering the entire nucleus with five iterations; the bleaching time was less than 1 s. The fluorescence of the ROI was collected in the Zen 2.1 software and exported to Excel for analysis.

### Protein extraction and Western blotting

Cells were washed with PBS prior to adding RIPA lysis buffer (Millipore; 20–188) supplemented with protease inhibitor (Roche; 11836170001) and phosphatases inhibitors 2 and 3 (SIGMA; P5726 and P0044, respectively). Cells were transferred to Eppendorf tubes and incubated 30 min on ice with regular vortexing. Cells were centrifuged at 16,000 × g and 4°C during 15 min, supernatants were collected and protein concentration was quantified using Protein Assay Dye Reagent Concentrate (Bio-Rad; 5000006). For subcellular fractions, cells were removed by scraping and suspended in lysis buffer containing 10 mM HEPES, 10 mM KCl, 0.1 mM EDTA, 0.1 mM EGTA, 1 mM DTT, PMSF in addition to protease and phosphatase inhibitors. Cells were incubated on ice for 15 min, 10% Igepal was added and cells were centrifuged for 30 s at 13,000 × g. The supernatant containing the cytoplasmic fraction was harvested. The pellet was resuspended in a buffer containing RIPA cell lysis buffer (1X), 330 mM NaCl, 0.5 mM PMSF and protease and phosphatase inhibitors, and was sonicated for a total duration of 5 min (cycles of 15 s with intervals of 15 s). The lysate was incubated on ice for 30 min and centrifuged for 30 min at 13,000 × g, and the supernatant containing the nuclear fraction was harvested. Equal amounts of proteins were prepared with laemmli buffer, denaturated and loaded on SDS/PAGE gel, then transferred to nitrocellulose membranes, and blocked with either 5% of milk or 5% of BSA according to the manufacturer’s instructions for each antibody. Membranes were incubated with primary antibody overnight at 4°C, and incubated with horseradish peroxidase-conjugated secondary antibodies (Jackson ImmunoResearch Laboratories). Protein bands were detected using Western Lightning Ultra (Perkin Elmer) and visualized using ImageQuant LAS 4000 (GE Healthcare). The Western blot data were quantified using ImageQuantTL software. Proteins of interest were normalized to *ß*-actin. To ensure the purity of the nuclear fraction, HDAC1 and Histone 3 were used as controls. Likewise, to ensure the purity of the cytoplasmic fraction, Tubulin was used as a control.

### siRNA transfection

6.5 × 10^5^ MDA-MB-231 and MDA-MB-468 cells were plated in 6-well plates. 24 h later, cells were transfected with siRNAs using Dharmafect #4 (Dharmacon), following the manufacturer’s instructions. CRP2 or SRF knockdown was achieved using optimized Flexitube GeneSolution siRNAs (Qiagen), while non-targeting siRNAs were used as controls (Eurogentec). 68 h later, cells were lysed and RNAs or proteins were extracted as previously described. The sequences of siRNA targets are indicated in Supplementary Table S1.

### *In vitro* pulldown assay

CRP2-GFP- and GFP-expressing MDA-MB-231 cells were plated in Petri dishes Ø 10 cm. Once cells reached 80% confluency, they were lysed with 10 mM Tris-HCl (pH 7.5), 150 mM NaCl, 0.5 mM EDTA, 0.5% NP-40, and 1× Halt protease inhibitor cocktail mix lysis buffer (Merck; 11836170001). The lysates were clarified by centrifugation at 16,000 × g for 15 min, and 1 mg of protein from each cell line were subjected to anti-GFP pulldown using GFP-TRAP magnetic agarose beads (ChromoTek), according to the manufacturer’s instructions.

### Proximity ligation assay

Duolink^®^ Proximity ligation assay (PLA) was performed on MDA-MB-231 cells expressing CRP2-GFP and transfected with FLAG-hSRF expressing plasmid (Addgene; 78343). After 24 h, cells were fixed with 4% PFA and permeabilized with 0.1% Triton-X100 and subsequently subjected to PLA analysis. The primary antibodies used are listed in Supplementary Table S2. Anti-mouse MINUS and anti-rabbit PLUS probes, in addition to the red detection reagent, were used according to the manufacturer’s instructions (Sigma Aldrich; DUO92008-30RXN). DAPI was added in the last washing step before mounting samples with Fluoromount (Sigma Aldrich; F4680). The number of PLA foci per cell were quantified using the Find maxima function of ImageJ software. As a control condition to evaluate unspecific PLA signal, the anti-FLAG antibody was omitted. Four independent experiments were conducted, including a total of at least 77 randomly selected cells.

### *In silico* promoter analysis

Genomic coordinates for *MMP-9* and *MMP-13* gene were extracted from NCBI database according to the human GRCh38 reference genome. Respective proximal promotor region spanning from −1,000 bp to +200 bp relative to the transcriptional start site (TSS) were retrieved from Eukaryotic Promoter Database (EPD) (Dreos et al., 2017). Prediction of regulatory promoter elements was performed using YAPP Eukaryotic Core Promoter Predictor tool (http://www.bioinformatics.org/yapp/cgi-bin/yapp.cgi; last accessed July 2022). Reported are promoter elements with a position-weight-matrix (PWM) similarity score to respective promoter element ≥0.8 within a region ranging from −50 bp to +50 bp relative the TSS. Putative synergistic combinations of promoter elements were also retrieved from YAPP Eukaryotic Core Promoter Predictor. The SRF PWM MA0083.2 was downloaded from JasparCore2022 Vertebrates database (Castro-Mondragon et al., 2022). Prediction of SRF binding motifs within the region −1,000 bp to +1 bp relative to the TSS of has been performed using the Find Individual Motif Occurrences discovery tool (FIMO, Version 5.4.1; (Grant et al., 2011)) of the collection of Motif-based sequence analysis tools (MEME suite; (Bailey et al., 2015)). Position, log-odd score, *p*-value, q-value and sequence of each predicted SRF binding motif are displayed in Supplemetary Table S3 and Figure 5A.

### Plasmids

The CRP2-GFP and CRP3-GFP expressing plasmid has been described previously (Hoffmann et al., 2014; Hoffmann et al., 2016; Hoffmann et al., 2018). N-terminus-CRP2-GFP and C-terminus-CRP2-GFP expressing plasmids were created by amplifying the coding sequence for amino acids 1-90 and 91-194, respectively, and inserting the ORF into the pEGFP-N1 vector using BamHI and XhoI restriction sites. The sequence of the related primers is given in Supplementary Table S1. Plasmids were transfected into cells using Lipofectamine 2000 (Thermo Fisher) according to the manufacturer’s instructions.

### Gene set enrichment analysis (GSEA)

GSEA was performed using clusterProfiler (R package, v4.4.4) (Wu et al., 2021) with “c2. all.v7.0. symbols.gmt” as a reference gene set. Genes were ranked according to differential expression analysis results (-log10 (FDR) * sign of log_2_FC) and the number of permutations was set to 10^6^. Gene sets with q-value <0.05 were called enriched. For visualization purpose, enriched gene sets describing close biological processes were aggregated into metagene sets. Q-values of the metagene sets were estimated by the geometric mean of single gene set q-values.

### Survival analysis

Kaplan-Meier survival plots, hazard ratio with 95% confidence intervals and log-rank *p* values were generated using the Kaplan-Meier Plotter tool (Gyorffy, 2021) to test for association between SRF expression and overall or distant metastasis free survival. Median values were used to define CRP2 high and low expression subpopulations. JetSet best probe set were selected for SRF and CRP2 expression, and patients classified as basal subtype according to PAM50 were selected. Gene expression data and survival information originate from GEO, EGA and TCGA.

### Statistical analysis

All graphs are shown as means ± SEM of the indicated number of independent experiments. For statistical comparison of different groups, Student *t-*test was used to determine significance. *p* values less than 0.05 were considered statistically significant.

## Data Availability

The datasets presented in this study can be found in online repositories. The names of the repository/repositories and accession number(s) can be found below: https://www.ncbi.nlm.nih.gov/geo/, GSE199822.

## Supplementary material

SUPPLEMENTARY FIGURE S1 CRP2 localizes to both the cytoplasmic and the nuclear compartments of MDA-MB-231 and MDA-MB-468 breast cancer cells. **(A)** Subcellular distribution of GFP-fused CRP2 in MDA-MB-231 breast cancer cells as visualized by confocal microscopy. Ventral and nuclear optical sections are shown (upper and lower panels, respectively). F-actin and nucleus are labeled using fluorescent phalloidin (red) and DAPI (blue). **(B)** Subcellular distribution of endogenous CRP2 in MDA-MB-468 breast cancer cells as visualized by immunofluorescence and confocal microscopy. Ventral and nuclear optical sections are shown (upper and lower panels, respectively). F-actin and nucleus are labeled using fluorescent phalloidin (red) and DAPI (blue). **(C)** Total (T), cytoplasmic (C) and nuclear (N) distribution of endogenous CRP2 in MDA-MB-468 breast cancer cells as evaluated by subcellular fractionation and western blot analysis. Histone3 (H3) and α-Tubulin are used as nuclear and cytoplasmic markers, respectively. Bars=20 µm. The microscopy images presented are representative of the entire cell population.

SUPPLEMENTARY FIGURE S2 CRP2 C-terminal domain slows down CRP2 shuttling to the nucleus. The nucleus of GFP, and GFP-fused full length CRP2, CRP2 C-terminal domain (Ct-CRP2-GFP) and CRP2 N-terminal domain (Nt-CRP2-GFP) expressing MDA-MB-231 cells were subjected to photo-bleaching and fluorescence recovery was followed for ten minutes. **(A)** Representative confocal microscopy images showing fluorescence before bleaching, immediately after bleaching, and after 9 minutes of recovery. **(B)** The average fluorescence recovery values over a 10-minute period with SEM for each time point. Pre-bleched values were normalized to 100%, and the bleached values were normalized to 0%. The experiment was performed on 16 cells for each condition. Bars=20 μm. **(C)** Total (T), cytoplasmic (C) and nuclear (N) distribution of endogenous CRP2 in MDA-MB-231 cells treated with leptomycin B for 16 hours (Leptomycin B) or mock-treated as a control (CTR). Histone3 (H3) and α-Tubulin are used as nuclear and cytoplasmic markers, respectively. CMR1/Exportin 1 is used as a conrol for letpomycin treatment. The blot presented is representative of three independent experiments.

SUPPLEMENTARY FIGURE S3 Ridgeplot (expression distribution) of selected genesets among the top enriched. The distribution of core genes within each geneset is shown. Positive distribution reflects upregulation of the geneset under CRP2 knockdown while negative distribution reflects downregulation of the geneset under CRP2 knockdown.

SUPPLEMENTARY FIGURE S4 Effects of CRP2 knockdown on the expression of other CRP family members. MDA-MB-231 cells were transfected with non-targeting control siRNAs (siCTR) or two different CRP2-targeting siRNAs (siCRP2#a and #b). Western blot analysis was performed to assess the expression levels of CRP2 , **(A)** CRP1 **(B)** and CRP3 **(C)** in the lysates of transfected cells. As a positive control for the anti-CRP3 antibody, cells were transfected with a plasmid allowing the expression of GFP-fused CRP3 were used. The blot images presented are representative of three independent experiments.

SUPPLEMENTARY FIGURE S5 Actin depolymerization promotes CRP2 nuclear translocation and increases MMP-9 and MMP-13 transcript levels in MDA-MB-468 cells. **(A)** Effects of control (DMSO) and cytochalasin-D (CD) treatment on F-actin-based structures and nuclear abundance of endogenous CRP2 in MDA-MB-468 cells as visualized by phalloidin staining (red) and immunofluorescence staining (green), respectively. Nuclei are labeled with DAPI (blue). The microscopy images presented are representative of the entire cell population. **(B)** Effects of control (DMSO) and cytochalasin-D (CD) treatment on endogenous CRP2 subcellular distribution in MDA-MB-468 cells as evaluated by subcellular fractionation and western blot analysis. Histone3 (H3) and α-Tubulin are used as nuclear and cytoplasmic markers, respectively. **(C)** CRP2 nuclear/cytoplasmic ratio quantified from four independent western blot analyses, similar to the one shown in (B). **(D,E)** MMP-9 (D) and MMP-13 (E) transcript levels in control and CD-treated cells. Results are expressed as the average ± SEM of at least three independent experiments (open circles); * denotes a *p*-value less than 0.05, *** a *p*-value less than 0.001 (two-tailed Student’s t test).

SUPPLEMENTARY TABLE S1 List and sequence of the siRNAs and primers used in the study.

SUPPLEMENTARY TABLE S2 List and reference of the antibodies used in the study.

SUPPLEMENTARY TABLE S3 Predicted regulatory elements and SRF binding motifs in human *MMP-9* and *MMP-13* promoters. Genomic coordinates of MMP9 and MMP13 according to CRCh38 reference. Regulatory promoter elements including initiator (INR), TATA-box (TATA) and downstream promoter elements (DPEs). SRF binding motif prediction based on the SRF matrix MA0083.2. Scores denote the log-odd scores. Positions are expressed relative to the respective TSS (+1). RNAseq data set (.xlsx): First leaflet: Raw RNA-seq count data. Second leaflet: Differential expression analysis (DEA-edgeR). Third leaflet: Gene Set Enrichment Analysis (GSEA) with a *“core enrichment”* column showing the genes contributing the most to the enrichment of the category. Fourth leaflet: Metagenesets used to create the bar plot in Figure 1G and the Ridgeplot in Supplementary Figure S3.
