# Supplementary figures and images for: Actin cytoskeleton depolymerization increases matrix metalloproteinase gene expression in breast cancer cells by promoting translocation of cysteine-rich protein 2 to the nucleus
Source: Front Cell Dev Biol. 2023 May 15;11:1100938. doi: 10.3389/fcell.2023.1100938 (PMC10229898; doi:10.3389/fcell.2023.1100938)

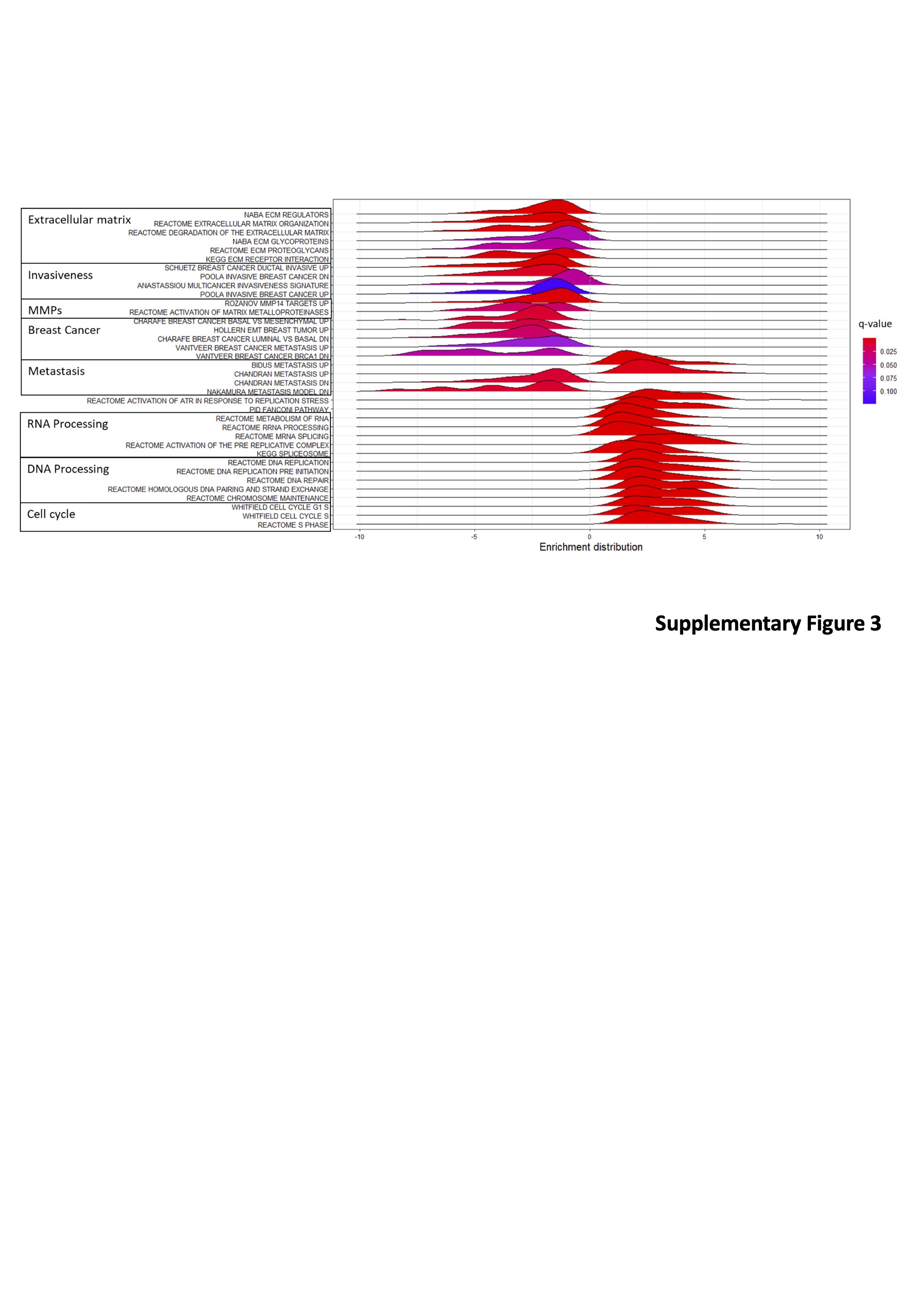

Supplement: Supplementary file 1 [file Image3.JPEG]

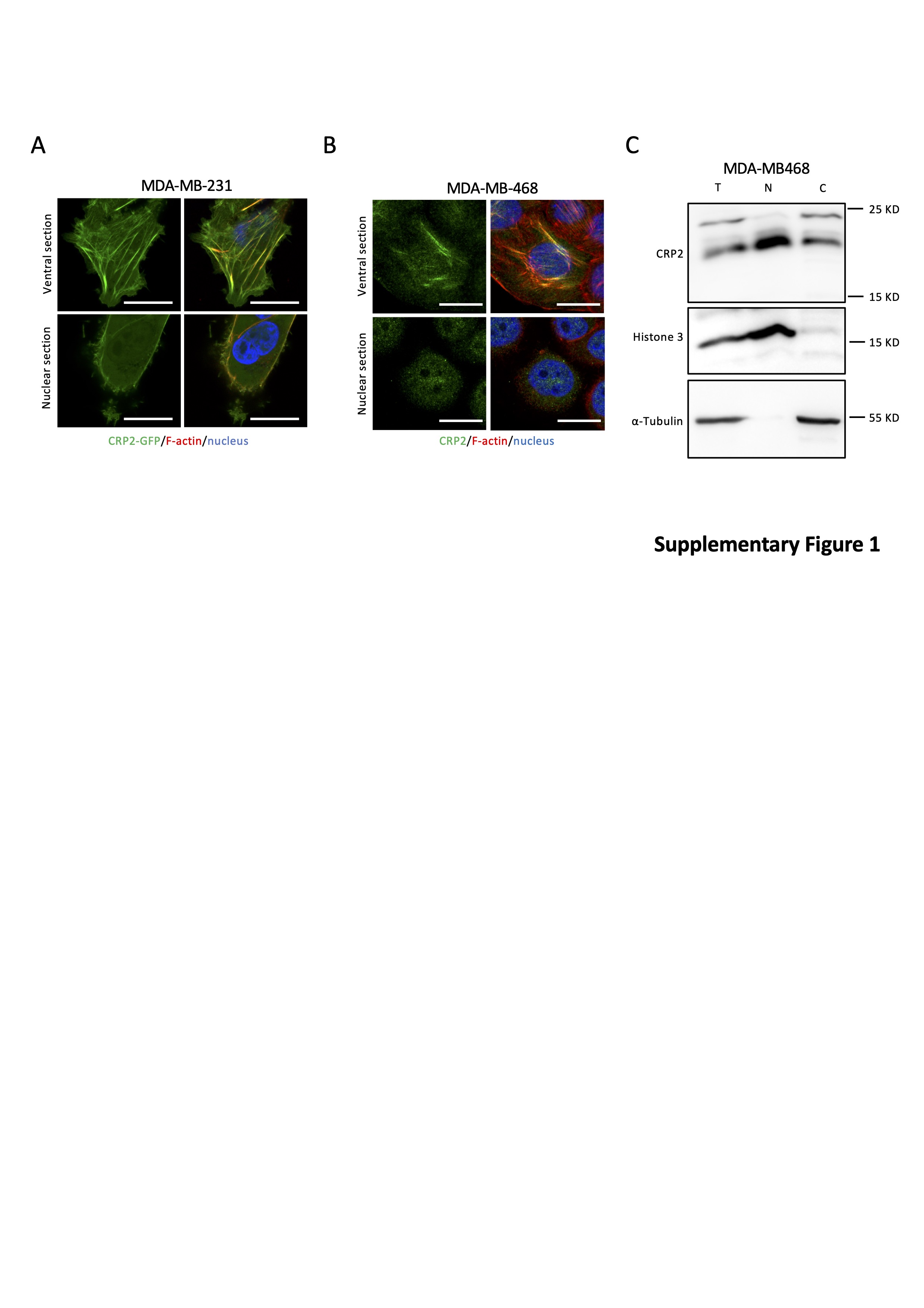

Supplement: Supplementary file 2 [file Image1.JPEG]

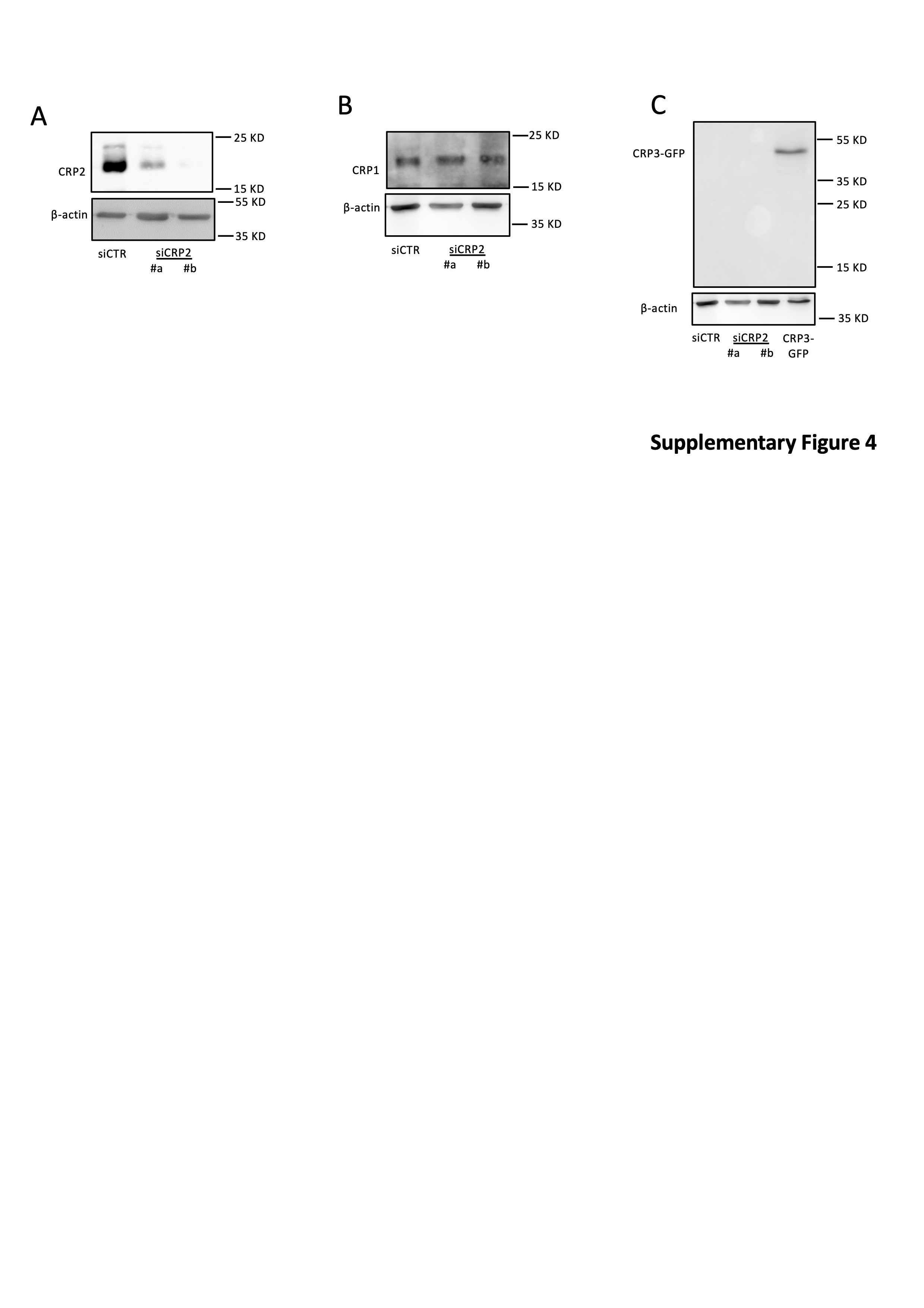

Supplement: Supplementary file 3 [file Image4.JPEG]

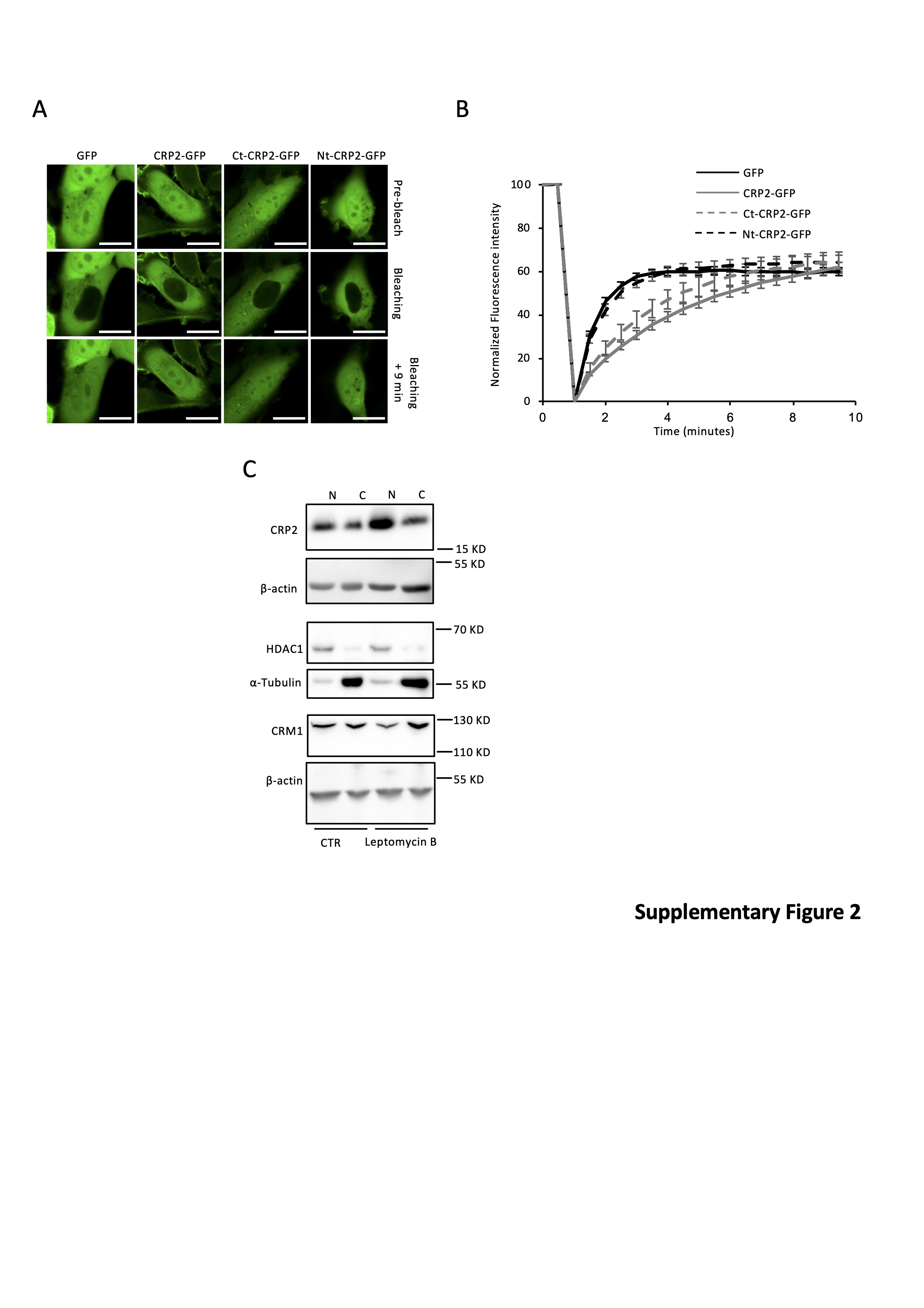

Supplement: Supplementary file 4 [file Image2.JPEG]

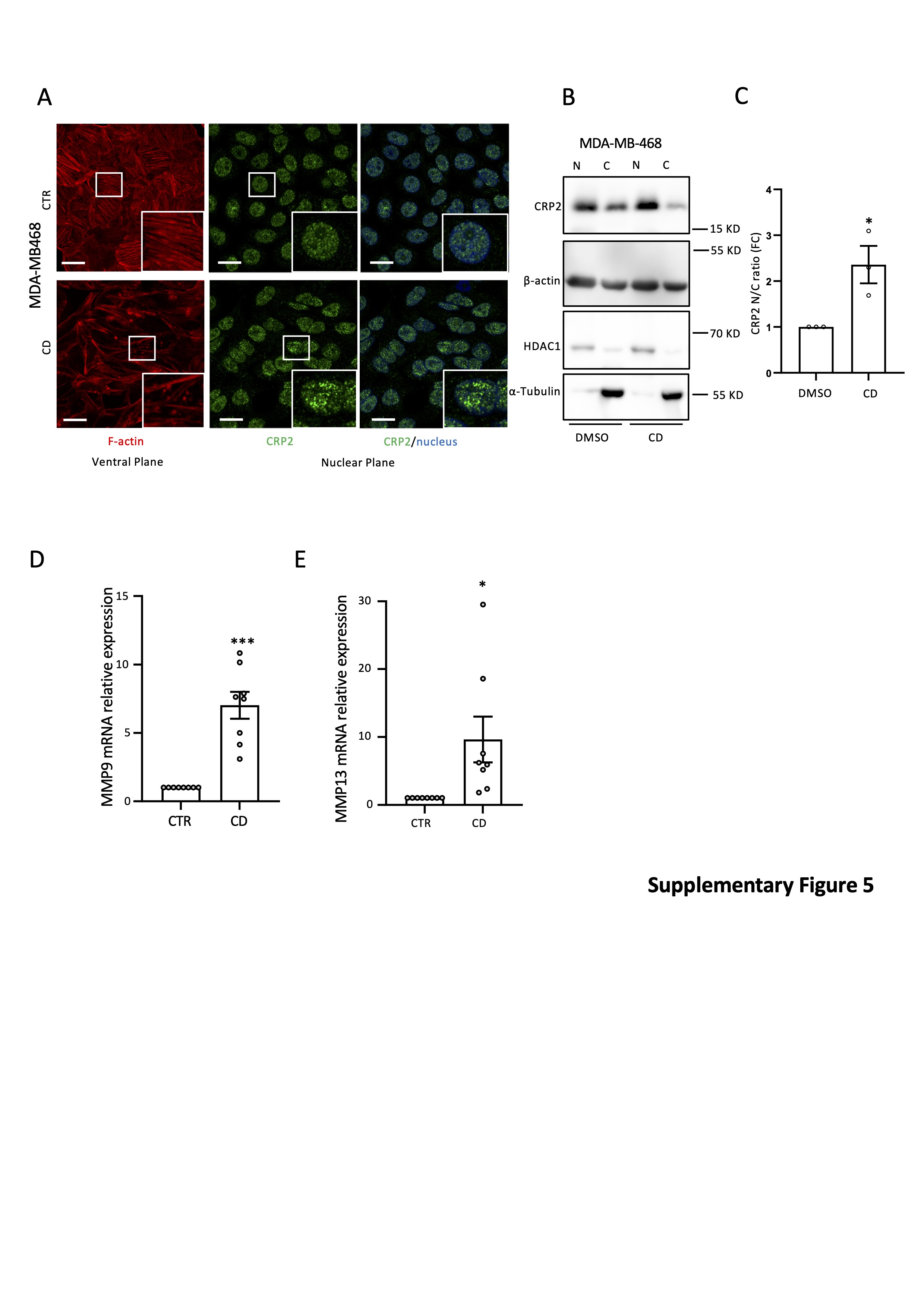

Supplement: Supplementary file 5 [file Image5.JPEG]
